# Current Perspective on Orthobiology Applications for the Treatment of Intervertebral Disc Degeneration (IDD)—A Narrative Review

**DOI:** 10.3390/medicina62040758

**Published:** 2026-04-15

**Authors:** Gianluca Conza, Maria Consiglia Trotta, Chiara Mastronardi, Alfonso Nocera, Annalisa Itro, Gabriele Martin, Gabriella Toro, Caterina Claudia Lepre, Marina Russo, Giuseppe Toro

**Affiliations:** 1 Multidisciplinary Department of Medical, Surgical and Dental Sciences, University of Campania “Luigi Vanvitelli”, 80138 Naples, Italy; gianluca.conza@studenti.unicampania.it (G.C.); chiara.mastronardi@studenti.unicampania.it (C.M.); alfonso.nocera@studenti.unicampania.it (A.N.); annalisa.itro@unicampania.it (A.I.); gabriele.martin@unicampania.it (G.M.); 2 Department of Experimental Medicine, University of Campania “Luigi Vanvitelli”, 80138 Naples, Italy; mariaconsiglia.trotta2@unicampania.it; 3 Unit of Radiology, San Paolo Hospital, 80125 Naples, Italy; gabriella.toro@tiscali.it; 4 PhD Course in Translational Medicine, University of Campania “Luigi Vanvitelli”, 80138 Naples, Italy; caterinaclaudia.lepre@unicampania.it; 5 PhD Course in National Interest in Public Administration and Innovation for Disability and Social Inclusion, Department of Mental, Physical Health and Preventive Medicine, University of Campania “Luigi Vanvitelli”, 80138 Naples, Italy; marina.russo@unicampania.it

**Keywords:** orthobiology, spine, intervertebral disc degeneration, low back pain, regenerative medicine, mesenchymal stem cells, PRP

## Abstract

*Background and Objectives*: Low back pain (LBP) is a leading cause of disability worldwide and is frequently associated with intervertebral disc degeneration (IVDD). Current therapeutic strategies are primarily symptomatic and do not restore native disc biology, largely due to the avascular nature of the intervertebral disc and the hostile inflammatory and mechanical microenvironment that characterizes degeneration. The aim of this study is to provide an updated and clinically oriented overview of the pathophysiology of IVDD and to evaluate the current evidence on mesenchymal stem cells (MSCs) and platelet-rich plasma (PRP)-based therapies. *Materials and Methods*: A focused narrative literature review was performed to evaluate current evidence on MSC- and PRP-based therapies for intervertebral disc degeneration (IVDD). The search was conducted in PubMed. Only studies in English were considered eligible. *Results*: Mesenchymal stem cells (MSCs) demonstrated regenerative and immunomodulatory effects primarily through paracrine mechanisms, enhancing extracellular matrix synthesis and reducing inflammation and apoptosis. MSC-derived extracellular vesicles emerged as a promising cell-free alternative, potentially overcoming limitations related to cell survival and safety. Platelet-rich plasma (PRP) showed anabolic and anti-inflammatory properties, promoting disc cell proliferation and matrix production, particularly in early-stage degeneration. Clinical studies, including randomized trials, reported significant improvements in pain and function for both MSC and PRP therapies, with favourable safety profiles. However, heterogeneity in treatment protocols and limited long-term data remain significant limitations. Orthobiologic therapies represent a minimally invasive option for patients with discogenic low back pain refractory to conservative treatment. Patient selection is crucial and should consider degeneration stage, disc viability, and clinical presentation. PRP is primarily indicated in early-stage degeneration (Pfirrmann II–III), whereas MSC-based therapies may be considered in selected patients with more advanced but still viable discs. Based on current evidence, a stepwise approach is proposed, progressing from conservative management to PRP, MSCs, and ultimately surgery. Orthobiologics should be integrated within a multimodal strategy including rehabilitation. *Conclusions*: MSCs and PRP represent a promising and, eventually, complementary orthobiologic therapies for IVDD. PRP is primarily effective in early degenerative stages as a biologic stimulator, whereas MSCs may provide regenerative benefits in more advanced but still viable discs. Further studies are necessary to standardize protocols and confirm long-term efficacy and safety.

## 1. Introduction

**Figure 1:**
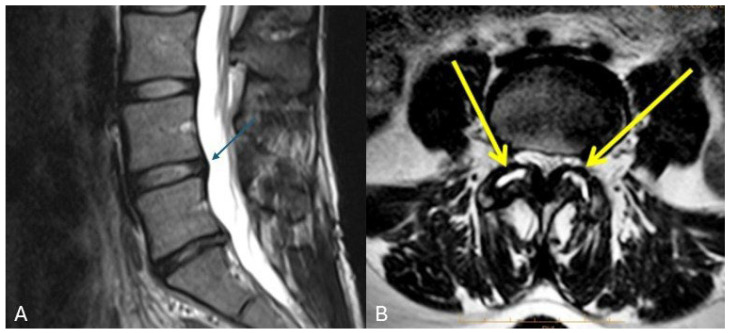
(**A**) MRI of a patient with discogenic low back pain. Note the L5-S1 discal herniation (blue arrow) associated with the discal degeneration as exemplified by the reduced signal on the T2-weighted imaging; (**B**) another patient’s MRI reporting a marked facet arthritis (yellow arrows).

Low back pain (LBP) is one of the most prevalent musculoskeletal disorders worldwide, representing a major contributor to disability and ranking among the highest contributors to healthcare-related economic burden [1]. Approximately 60–80% of individuals will experience LBP at some point during their lifetime [2]. Furthermore, recent projections suggest that the global prevalence of LBP will continue to increase, potentially affecting up to 890 million people by 2050 [3]. Although most instances of LBP are related to extra-spinal disorders, degenerative changes in the spine—primarily involving the intervertebral discs and the facet joints (facet arthritis)—are frequently the main causes of this issue (Figure 1) [4]. Current treatment strategies, including surgical procedures such as decompression and fusion, as well as pharmacological therapies, are largely aimed at symptom control rather than restoration of native disc structure or function [5]. However, treatments capable of restoring spinal degenerated structures are not currently available in clinical practice [4]. This limitation may be explained by the inherently poor regenerative capacity of the intervertebral disc (IVD), which is characterized by low cellularity and a lack of direct vascular supply [5]. Moreover, the biomechanical environment of the spine—comprising cyclic loading, hydrostatic pressure, and mechanical stress gradients—further complicates the reparative processes [6]. Consequently, degenerative changes often progress irreversibly, culminating in chronic pain syndromes, deformity, and neurological compromise [7].

For these reasons, researchers are increasingly investigating potential alternatives to conventional therapeutic strategies, including targeted injection-based approaches and spinal mesotherapy [8]. Regenerative medicine has emerged as one of the most promising recent trends in healthcare, owing to its broad range of potential applications across multiple medical fields [9]. Regenerative medicine proposes a novel paradigm that aims to replace, restore, or regenerate damaged tissues through biological and engineering principles [10,11]. In orthopedic surgery, regenerative medicine has been extensively investigated, particularly for the treatment of osteoarthritis [12,13,14,15,16]. An increasing body of evidence suggests that it may represent a valuable therapeutic option for reducing pain and improving function in several orthopaedic and traumatic conditions [12].

In the context of spinal pathology, regenerative approaches have been demonstrated as promising tools to treat intervertebral disc degeneration, spinal cord injury (SCI), vertebral bone defects, and post-fusion non-unions [17,18].

Over the last decade, translational progress in regenerative spinal therapies has accelerated, owing to advancements in stem cell biology, scaffold design, and controlled drug delivery systems [19]. Preclinical models using mesenchymal stem cells (MSCs) derived from bone marrow, adipose tissue, and umbilical cord have shown the capacity to differentiate into chondrocytic, osteogenic, and neural phenotypes when exposed to specific microenvironmental cues [17,18]. Concurrently, bioengineered scaffolds and hydrogels—often composed of collagen, hyaluronic acid, or polyethylene glycol—have been optimized to mimic the viscoelastic and biochemical properties of the native ECM [20,21,22]. These systems support cell adhesion, proliferation, and matrix deposition, which are essential for the reconstitution of functional spinal tissues [22].

Intradiscal MSC injections have demonstrated improvement in pain scores and disc hydration on MRI in patients with degenerative disc disease [23,24]. Similarly, in vertebral fractures and spinal fusion procedures, tissue-engineered bone graft substitutes incorporating osteoprogenitor cells and growth factors such as bone morphogenetic protein-2 (BMP-2) have shown enhanced osteointegration and fusion rates compared to conventional allografts [25,26].

However, despite encouraging progress, regenerative spinal medicine remains constrained by several translational barriers. The spinal microenvironment’s immunological hostility, characterized by pro-inflammatory cytokine release and fibrosis, limits graft survival and differentiation potential [27]. In addition, ethical considerations regarding stem cell sources, risks of tumorigenicity with induced pluripotent stem cells (iPSCs), and heterogeneity of clinical trial methodologies pose significant regulatory challenges [28]. Thus, ongoing research is focused on refining the cellular and molecular precision of regenerative therapies, developing standardized delivery systems, and enhancing long-term safety and functional outcomes [29].

The purpose of this review is to provide an up-to-date overview of the biological rationale and results of MSC- and PRP-based therapies for IVDD.

## 2. Materials and Methods

An up-to-date narrative literature review to January 2026 was performed to evaluate current evidence on orthobiologic therapies for intervertebral disc degeneration (IVDD), with a specific focus on mesenchymal stem cells (MSCs) and platelet-rich plasma (PRP).

The structured search was conducted in PubMed by two independent reviewers Search terms combined keywords related to disc degeneration (“intervertebral disc degeneration,” “discogenic low back pain,” “degenerative disc disease”) with regenerative strategies (“mesenchymal stem cells,” “MSCs,” “bone marrow aspirate concentrate,” “adipose-derived stem cells,” “platelet-rich plasma,” “PRP,” “platelet-rich fibrin,” “intradiscal injection,”). Relevant studies for our multidisciplinary discussion were included by consensus between reviewers (C.M. and A.N.). Controversies between the two reviewers were analyzed by a third author for the final inclusion decision (G.T.).

The literature search was conducted using only the PubMed database because, according to Rollin et al., 90% of high-quality studies can be retrieved from this database [15]. Therefore, most of the relevant literature could be retrieved on it, and PubMed research should be considered as cost-effective to summarize the knowledge around a specific topic [15,16]. Data from each retrieved study were collected using a pre-arranged form and discussed by the research team.

Eligible studies were published in English and included in vitro, animal, and clinical investigations assessing the biological mechanisms or therapeutic effects of MSCs or PRP in IVDD. Randomized controlled trials, prospective and retrospective cohort studies, and relevant translational research were considered. Case reports, editorials, and studies not primarily focused on disc degeneration were excluded.

**Figure 2:**
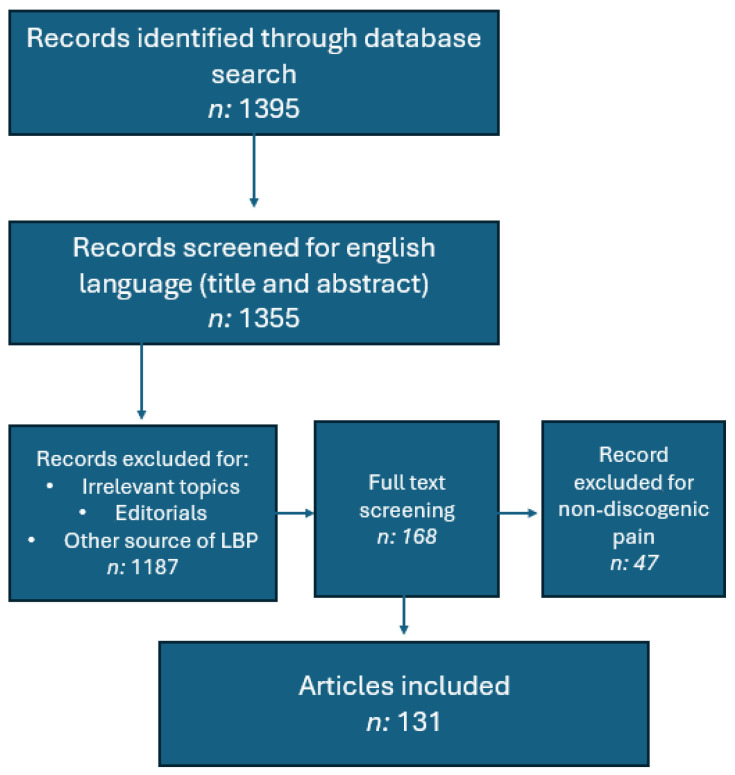
Flow diagram of the literature selection process.

The selection process involved initial screening of titles and abstracts, followed by a full-text evaluation of potentially relevant articles (Figure 2). Given the narrative nature of the review, no formal risk-of-bias assessment or quantitative synthesis (meta-analysis) was conducted. Instead, the available evidence was qualitatively synthesized and organized into thematic sections, including pathophysiology, MSC-based therapies, PRP-based therapies, and clinical applications. Our study followed the 2018 Academy of Nutrition and Dietetics Narrative Review Checklist [30].

## 3. Anatomy and Development of Intervertebral Disc

The intervertebral disc is the largest avascular environment in the body and is subjected to the most extensive degenerative changes in the human body [31]. The human spine comprises a total of 23 IVDs: 6 cervical, 12 thoracic, and 5 lumbar discs, accounting for about a quarter of the total height of the spine [32]. IVDs lie between the vertebral bodies and are separated from them by a hyaline cartilage endplate [33]. Each IVD has three distinct anatomical regions: the central nucleus pulposus (NP), the fibrocartilaginous annulus fibrosus (AF), and, superiorly and inferiorly, two cartilaginous endplates (CEP) [32]. The major components of IVDs originate from two embryonic tissues: the NP from the notochord and the AF from the sclerotome of the somites [34]. The nucleus pulposus contains high levels of aggrecan that attracts water and permits it to absorb external forces and dissipate them uniformly to the surrounding anulus fibrosus [35]. In humans, the notochord cells die within the first few years of life, accompanied by a gradual decrease in the water content of the nucleus pulposus [35]. The NP contains randomly oriented collagen fibres and radially arranged elastin fibres, which can reach lengths of up to 150 mm [36]. These fibres are embedded in a highly hydrated gel rich in aggrecan [36]. Chondrocyte-like cells are observed throughout the matrix but at a low density (approximately 5000 cells/mm^3^), and occasionally enclosed within a capsular structure [36]. Surrounding the nucleus pulposus lies the AF with a sharp boundary with the NF in young individuals (<10 years of age) [36]. During growth and skeletal maturation, the boundary becomes less defined, and with ageing, the nucleus becomes more fibrotic and less gel-like [36]. The compartment of the sclerotome that will form the AF of the IVD can be traced back to the somitocoel cells of the somite [34]. Somites are transient embryonic structures that consist of an outer epithelial sphere with a central core of mesenchymal cells called the somitocoel [34]. The cells in the developing AF are fibroblast-like and elongate to organize into concentric circles [34]. This orientation is caused by the organization of an intracellular network of actin stress fibres and is thought to provide a template for the deposition of collagen, responsible for the lamellar structure of the AF [34]. The developing AF consists of two components: an inner fibrocartilaginous portion (iAF) and a more fibrous outer portion (oAF) [37]. The Pax1/9 genes were shown to promote both iAF and oAF expression profiles in differentiating cells; however, Pax1/9 in combination with TGFß and BMP was primarily involved in promoting iAF development [38]. The transcription factors Pax1 and Pax9, which belong to the same subfamily of Pax genes, encode proteins containing a highly conserved DNA-binding domain known as the paired box [38]. These factors are co-expressed in, and are essential for, the development of sclerotome-derived vertebral bodies and IVD [38]. With further differentiation, Pax 1 and Pax2 expression progressively decreases within the iAF while remaining in the oAF [38]. The inner part of the AF initially differentiates as hyaline cartilage and accumulates glycosaminoglycans and type II collagen [39]. On the other hand, the outer part differentiates into fibroblastic tissue, which becomes organized into oriented laminae of cells that secrete type I collagen [39].

During growth and especially with the acquisition of upright posture, the outer portion of the anulus undergoes a gradual fibrocartilaginous metaplasia, characterized by an increase in glycosaminoglycans (GAGs) and type II collagen, resembling the inner part [39]. Fibrocartilaginous metaplasia represents an adaptive mechanism in response to increasing mechanical load. In this process, the TGF-β1 factor plays a fundamental role [39].

With ageing and degeneration, the IVD undergoes progressive morphological changes and becomes increasingly disorganized [36]. The anular lamellae often become irregular, and the collagen and elastin networks lose their organized structures [36]. Degeneration is frequently associated with IVD fissurization along with an increased ingrowth of nerves and blood vessels, also in unusual areas [40]. Certain cells attempt to mitigate the damage through aberrant proliferation, resulting in the formation of clusters; however, these reparative efforts are often disordered and functionally ineffective. Concurrently, it is estimated that upwards of 50% of the cell population within a degenerate disc undergoes cell death [40,41,42].

The mechanical function of the IVD is primarily determined by its extracellular matrix, whose composition and organization govern its response to load [36]. Mechanical function is mainly provided by collagen and aggrecan [36]. The collagen network [43] confers tensile strength and anchors the disc to bone, while aggrecan maintains tissue hydration through osmotic pressure generated by its glycosaminoglycan chains [44,45]. The matrix is highly dynamic, with continuous synthesis and degradation provided by metalloproteinases and aggrecanases [46]. The balance between these processes determines matrix integrity, mechanical performance, and the maintenance of the disc’s normally avascular and aneural state [46].

Among matrix metalloproteinases, the collagenase subgroup—comprising MMP-1, MMP-8, and MMP-13—is capable of cleaving the triple-helical domains of fibrillar collagens, specifically types I, II, and III [46]. The collagen fragments generated by this initial cleavage can subsequently be further degraded by gelatinases, notably MMP-2 and MMP-9, whose expression has been shown to increase in degenerated discs [46].

## 4. Pathophysiology of IVD

The degeneration of IVD is characterized by a progressive, cell-mediated breakdown of the IVD structure and function, driven by the aforementioned imbalance between anabolic and catabolic processes within the disc microenvironment [47]. Several studies have shown that multiple pathological factors such as inflammation, oxidative stress, mitochondrial dysfunction, and abnormal mechanical loading, are involved in the development of IVD degeneration (IVDD) [48,49,50]. Particularly, inflammatory mediators such as IL-1β disrupt the metabolic homeostasis of the ECM [51]. In NP degeneration, ECM catabolic enzymes (i.e., MMPs; a disintegrin and metalloprotease with thrombospondin motifs (ADAMTS)) present a high expression by resident cells, ultimately leading to ECM degradation [47,52,53]. In recent years, increasing evidence has demonstrated that mitochondrial dysfunction enhances the production of reactive oxygen species (ROS) and that excess ROS promotes IVDD by regulating matrix metabolism, pro-inflammatory phenotype, autophagy, senescence, and apoptosis of IVD cells [6,54,55,56]. Aberrant mitochondrial dynamics result in mitochondrial fragmentation, loss of membrane potential, and release of pro-apoptotic factors [57,58,59]. Defective mitophagy impairs the removal of damaged mitochondria, exacerbating cellular stress and perpetuating degeneration [60,61]. Mitochondrial dysfunction also activates inflammatory pathways, such as the cGAS-STING axis, through mtDNA leakage, amplifying local inflammation and catabolic signalling [62]. Downregulation of mitochondrial biogenesis and quality control mechanisms, including PGC-1α signalling, further impairs disc cell survival and ECM maintenance [59]. Collectively, these processes contribute to the loss of disc cell viability, increased apoptosis, and breakdown of the ECM, which are hallmarks of IVD degeneration [41]. Abnormal mechanical stress applied to the IVD promotes ECM degradation by IVD cells and accelerates the development of IVDD [63]. The process of IVDD also consists of pyroptosis, inflammation, and an acidic environment, involving the regulation of key genes such as NLRP3, ASIC1a, IL-1β, TNF-α, and GSDMD [64]. Particularly, emerging evidence suggests that acid-sensing ion channels (ASICs) (i.e., ASIC1a) may play a pivotal role in linking extracellular acidification to inflammasome activation and cell death in nucleus pulposus cells [27]. Activation of ASIC1a under acidic conditions promotes Ca^2+^ influx, mitochondrial dysfunction, and NLRP3 inflammasome assembly, leading to caspase-1 activation, gasdermin D (GSDMD) cleavage, and IL-1β release, thereby accelerating pyroptotic cell death and disc degeneration [64,65]. The NLRP3 inflammasome is a multiprotein complex that, when activated in IVD cells, drives the pathophysiology of disc degeneration through three main mechanisms: sensing and integrating diverse danger signals, promoting pyroptosis, and accelerating ECM breakdown [66]. Once activated, the NLRP3 inflammasome cleaves pro-caspase-1 to its active form, which in turn processes pro-IL-1β and pro-IL-18 into their mature, secreted forms. Caspase-1 also cleaves gasdermin D, forming membrane pores and inducing pyroptosis, a lytic and highly inflammatory form of programmed cell death [67]. Pyroptosis results in the release of inflammatory cytokines and cellular contents, amplifying local inflammation and tissue damage [63,68]. The inflammatory milieu and cell death mediated by NLRP3 activation drive extracellular matrix breakdown by upregulating matrix metalloproteinases and aggrecanases, suppressing anabolic matrix gene expression, and perpetuating a catabolic environment [69]. In summary, the NLRP3 inflammasome integrates multiple pathogenic signals in disc cells, induces pyroptosis, and promotes ECM degradation, thereby playing a central role in the progression of IVD degeneration [67,70,71].

Abnormal mechanical load contributes to IVD degeneration through a cascade of structural, cellular, and molecular events. Excessive or aberrant loading—whether through chronic overload, repetitive microtrauma, or instability—disrupts the disc’s extracellular matrix, leading to fissures in the annulus fibrosus, endplate damage, and loss of disc height [72]. This structural failure uncouples the local mechanical environment from normal load distribution, resulting in inappropriate cell responses and further matrix breakdown [72,73]. At a cellular level, abnormal load induces cell death (through both apoptosis and pyroptosis), senescence, and upregulation of proinflammatory cytokines (e.g., TNF-α, IL-1β, IL-6), with catabolic activity, accelerating proteoglycan and collagen degradation [55,63,74,75,76]. The loss of hydrophilic proteoglycans leads to disc desiccation and altered biomechanics, increasing annular stress and predisposing to fissuring and herniation [73,75].

Recent molecular insights have shown that abnormal stress can trigger epigenetic modifications (e.g., WTAP/YTHDF2-dependent m6A methylation of TIMP3), further enhancing matrix metalloproteinase activity and extracellular matrix degradation [51]. Additionally, abnormal loading induces abnormal nerve ingrowth and inflammation, contributing to pain and further degeneration [74,76]. Overall, the IVDD pathophysiology is characterized by a vicious cycle: mechanical overload or instability initiates structural damage, which drives aberrant cell-mediated catabolic responses, perpetuating matrix breakdown and biomechanical dysfunction [63,73,77]. This cycle is amplified by inflammation, cell death, and molecular dysregulation, ultimately resulting in progressive disc degeneration [78]. Chronic inflammation further amplifies disc degeneration through a positive feedback loop between pro-inflammatory cytokines and ECM degradation [79]. Apart from the upregulation of catabolic enzymes, inflammatory signalling activates NF-κB and MAPK pathways, which not only exacerbate ECM breakdown but also promote senescence and apoptosis of disc cells [78,79,80,81,82]. MAPK pathways, specifically p38 MAPK and JNK, regulate the expression of some catabolic enzymes and pro-apoptotic factors associated with the ECM degradation and cell apoptosis [83,84]. The role of MAPK signalling is underlined by the observation that the inhibition of p38 MAPK and JNK, reducing MMP and ADAMTS expression, attenuates disc degeneration and apoptosis in disc cells [72,85,86]. Metabolic reprogramming has also been implicated in IVDD progression [87]. Degenerated discs exhibit a shift toward glycolytic metabolism, leading to lactate accumulation and further acidification of the disc microenvironment [64]. This acidic milieu reinforces ASIC-mediated signalling and inflammasome activation, forming another vicious cycle between altered metabolism, inflammation, and cell death [64,88].

## 5. Orthobiology in IDD

### 5.1. Stem Cell Therapy

The application of MSCs in IVDD seems promising [89]. In fact, after their application, exogenous MSCs migrate to the site of injury, where they differentiate into IVD-related cells and stimulate the proliferation of resident disc cells [90]. This activity increases cell density at the damaged site and contributes to disc repair [90]. In addition, MSCs promote IVD regeneration by inhibiting apoptosis and modulating the local immune microenvironment [91]. Despite these promising effects, some limitations, like tumorigenic potential, fibrosis, injection-related toxicity, immune rejection, and limited differentiation capacity, may compromise the therapeutic effects of MSC-based treatments [92]. Consequently, increasing attention has been directed toward MSC-derived soluble mediators, particularly extracellular vesicles (EVs) and conditioned supernatants, as alternative therapeutic strategies in several clinical settings [11,93]. EVs are classified into three main categories—apoptotic bodies, microvesicles, and exosomes—based on their size, biogenesis, and molecular content [94]. Apoptotic bodies (50–4000 nm) are generated during the late stages of apoptosis and contain cellular organelles, membrane components, and nuclear materials [95]. In contrast, microvesicles (100–1000 nm) are shed directly from the plasma membrane of viable cells and exhibit heterogeneous morphology [94]. Exosomes are the smallest EVs (30–150 nm) and are formed through inward budding of the late endosomal membrane, resulting in multivesicular body formation [96]. MSC-derived exosomes have shown therapeutic potential in various degenerative diseases. In IDD treatment, exosome-based therapy represents a cell-free approach that avoids many of the limitations associated with stem cell transplantation [89]. Independently from the donor site, MSCs contribute to the treatment of IVDD by modulating immune responses, ECM synthesis, and secreting bioactive soluble factors [89]. MSC-based strategies for tissue repair can be categorized into two approaches: unmanipulated and manipulated. In the first, MSCs are directly injected into the injury site, where they differentiate in response to the local tissue microenvironment [97]. This approach carries the risk of differentiation into undesired cell lineages, potentially limiting therapeutic efficacy [97]. In the second approach, MSCs are firstly pre-differentiated into specific cell types using growth factors, differentiation cues, or gene-based in vitro techniques, and then applied to the site of interest [98]. These pre-differentiated cells generally exhibit a more stable phenotype and greater resistance to trans-differentiation within host tissues [98]. Nevertheless, pre-induction of MSC differentiation may compromise their immunomodulatory properties and ultimately may reduce treatment effectiveness [98]. Intravenous administration represents a possible route of injection in cell-based therapies. However, only a small fraction of injected cells ultimately reach the damaged tissue [89]. To improve cell retention, intradiscal injection of MSCs has been proposed for IDD treatment. However, intradiscal injection is still associated with some limitations, including cell leakage from the disc due to annular puncture [99]. Leaked cells may alter cartilage endplate (CEP) integrity and promote ectopic osteophyte formation, thereby impairing disc function [99]. The post-transplantation survival of MSCs within the IVD remains poorly defined, although evidence suggests that the majority of transplanted cells fail to survive [100]. This loss can result in the accumulation of necrotic debris and apoptotic cells, potentially disrupting disc homeostasis and negatively affecting therapeutic outcomes [100]. Experimental studies have demonstrated that MSCs rapidly disappear from target tissues within seven days following injection, irrespective of the delivery route [100]. Within 24 h of transplantation, MSCs exposed to the hypoxic and nutrient-limited disc environment activate hypoxia-responsive pathways, leading to caspase-3-mediated apoptosis [100]. These apoptotic cells are subsequently cleared by resident macrophages [100].

#### 5.1.1. Pre-Clinical Studies

Several studies suggest that MSC apoptosis plays a significant role in modulating both innate and adaptive immune responses, thereby influencing some of the therapeutic effects of MSC-based treatments [101]. The mechanisms governing MSC survival, function, and immunoregulatory activity within the complex IVD microenvironment—particularly for bone marrow-derived MSCs—remain incompletely understood, as most studies have primarily focused on transplantation outcomes rather than cell survivorship [101]. In vitro investigations showed that MSCs may produce regenerative effects on IVD (IVD) cells, particularly those of the nucleus pulposus (NP) and annulus fibrosus (AF) [102]. When co-cultured with degenerative human disc cells, MSCs induce differentiation toward an NP-like phenotype, evidenced by increased expression of key ECM-related genes, including COL2A1 (type II collagen), ACAN (aggrecan), and SOX9, which are pivotal for disc homeostasis and matrix synthesis [19,102]. Concurrently, MSCs attenuate disc catabolism by suppressing matrix-degrading enzymes such as MMP-3, MMP-13, and ADAMTS-5, while also reducing the expression of pro-inflammatory cytokines, including IL-1β and TNF-α, released by senescent or degenerative disc cells [103]. These regenerative effects are predominantly mediated through paracrine mechanisms [19]. In particular, EVs derived from MSCs—enriched with regenerative microRNAs such as miR-21, miR-155, and miR-199a—have been shown to enhance matrix synthesis and inhibit apoptotic and inflammatory pathways in NP cells [104]. Furthermore, studies using three-dimensional (3D) culture models that replicate the hypoxic disc microenvironment demonstrate that MSCs retain chondrogenic differentiation potential and viability under low oxygen conditions (1–5%) [105]. This adaptive capacity is associated with HIF-1α activation and enhanced autophagy, both mechanisms that support cell survival and extracellular matrix production [105]. Taken together, these in vitro findings suggest that MSCs might favourably modulate the degenerative disc microenvironment by rebalancing the anabolic–catabolic metabolism and controlling inflammatory signalling, thereby providing a strong rationale for their therapeutic application in IVD degeneration.

#### 5.1.2. Clinical Evidence

##### Case Reports and Meta-Analyses

Clinical studies and systematic reviews indicate that MSC therapy showed promising clinical efficacy in treating IDD. In 2023, a meta-analysis involving 245 patients demonstrated significant improvements in pain (mean Visual Analogue Scale reduction of 41.6 points) and disability (Oswestry Disability Index improvement of 22 points) with no major adverse events, suggesting that MSC therapy might be effective and safe for discogenic pain relief [106].

##### Observational Studies

A cohort study of 33 patients treated with allogenic umbilical cord-derived MSCs reported sustained pain reduction (VAS decrease of 6.6) and functional improvement (ODI improvement of 38.3%) at two-year follow-up, with 90% of patients showing good-to-excellent outcomes and no adverse reactions [107]. Despite these encouraging findings, observational designs are subject to confounding factors and lack randomization.

##### Interventional Studies (Randomized Controlled Trials)

More robust evidence has emerged from randomized controlled trials. A phase IIB double-blind RCT of autologous bone marrow-derived MSCs reported a statistically significant improvement in pain, quality of life, and work ability at 12 months compared with sham treatment, without serious adverse events [108]. Noriega et al. presented a long-term (≈42-month) follow-up of a randomized, controlled trial in patients with chronic lumbar degenerative disc disease who received intradiscal injections of allogeneic bone marrow-derived mesenchymal stem cells (MSCs), which demonstrated durable clinical benefits compared with controls [109]. Most importantly, those patients treated with MSCs maintained significant improvements in pain and function from the first year up to 3.5 years post-treatment without serious adverse events [109]. In contrast, control patients exhibited progression of disc degeneration on MRI over the same period, whereas the MSC-treated group showed a significant reduction in Pfirrmann degeneration grade, suggesting potential structural improvement [109]. These studies provide stronger evidence of efficacy; however, they remain limited by relatively small cohorts and variability in treatment protocols.

The available clinical evidence presents several strengths, including the presence of randomized controlled trials and consistent reporting of clinically meaningful improvements in pain and function. However, important limitations must be considered. Most studies are characterized by relatively small cohorts, heterogeneity in MSCs sources (bone marrow, adipose tissue, umbilical cord), variability in cell processing and dosing protocols, and limited long-term follow-up. Overall, while MSC-based therapies demonstrate promising regenerative potential and clinical benefit, the current evidence remains limited by methodological heterogeneity and insufficient long-term data.

Strength of evidence: moderate (promising clinical outcomes supported by early randomized trials but limited by heterogeneity and sample size).

See Table 1 for further details.

### 5.2. Platelet-Rich Plasma

Platelet-rich plasma (PRP) is an autologous blood derivative capable of releasing large quantities of α-granules rich in growth factors (GFs), promoting cell proliferation and differentiation, and it has been extensively applied in both oral surgery and orthopedics [110]. Platelets possess several types of secretory organelles, including dense granules, α-granules, and lysosomes [111]. Among these, the former are the majority, with each platelet containing approximately 50–80 granules of 200–500 nm [111]. α-granules store both membrane-associated and soluble proteins. The membrane-bound components include integrins, immunoglobulin superfamily receptors, and leucine-rich repeat family receptors [112]. Upon platelet activation, these membrane proteins are translocated to the platelet surface, while soluble proteins are secreted into the extracellular milieu. Notably, α-granules also harbour small vesicles known as exosomes [112], which can likewise be released during platelet activation. Proteomic analyses have demonstrated that activated α-granules secrete more than 300 distinct soluble proteins [113]. These bioactive molecules contribute to a wide range of biological processes, including hemostasis, inflammation, antimicrobial defence, angiogenesis, and tissue repair [113].

PRP has been classified into four categories based on leukocyte and fibrin content: pure PRP (P-PRP), leukocyte-rich PRP (L-PRP), leukocyte- and platelet-rich fibrin, and pure platelet-rich fibrin [114]. As PRP is derived from autologous plasma, it offers several advantages: (1) minimal risk of immune rejection, (2) avoidance of disease transmission, and (3) simple and cost-effective preparation by centrifugation, making it substantially less expensive than recombinant growth factors (GFs) [110]. PRP is primarily composed of growth factors, inflammatory cells, and adhesion molecules. The major GFs include platelet-derived growth factor, transforming growth factor-β (TGF-β), vascular endothelial growth factor (VEGF), and epithelial growth factor [12]. Accumulating experimental evidence has demonstrated that PRP enhances angiogenesis, stimulates cell proliferation, and increases collagen synthesis, thereby facilitating the repair of damaged tissues such as tendons, ligaments, cartilage, and other avascular tissues with limited intrinsic healing capacity [110]. These findings suggest that MSCs-based therapy may represent a promising option for patients with chronic discogenic low back pain who have failed first-line conservative treatment, particularly where a moderate disc degeneration is present.

#### 5.2.1. Pre-Clinical Studies

PRP therapy represents a promising biologically based intervention for IDD, thanks to the controlled release of autologous growth factors and cytokines that aid in restoring the catabolic–anabolic balance within the degenerated disc microenvironment [111]. The therapeutic rationale arises from the proved PRP’s ability to deliver concentrated platelets that, upon activation, release bioactive mediators such as platelet-derived growth factor (PDGF), transforming growth factor-β (TGF-β), vascular endothelial growth factor (VEGF), and insulin-like growth factor-1 (IGF-1), which collectively stimulate proliferation of nucleus pulposus (NP) and annulus fibrosus (AF) cells and enhance extracellular matrix (ECM) synthesis of proteoglycans and type II collagen [115]. PRP exerts anti-apoptotic and anti-inflammatory effects by suppressing NF-κB signalling and downregulating IL-1β and TNF-α expression, thereby reducing catabolic enzyme activity (MMP-3, ADAMTS-5) associated with disc matrix degradation [116]. Moreover, recent evidence indicates that PRP-derived exosomes enriched with microRNA-141-3p modulate the Keap1-Nrf2 antioxidant pathway, attenuating oxidative stress-induced pyroptosis and preserving NP cell viability [117]. These molecular and cellular mechanisms contribute to the restoration of disc homeostasis, improved nutrient microcirculation, and potential reversal of degenerative progression [110]. Collectively, PRP provides a multifactorial regenerative stimulus that targets the core pathological processes of IDD—cellular senescence, inflammation, and matrix breakdown—supporting its use as a minimally invasive biologic therapy for discogenic pain and degeneration [111].

Akeda et al. showed that PRP stimulates porcine nucleus pulposus (NP) and annulus fibrosus (AF) cell proliferation and enhances proteoglycan and collagen synthesis, while platelet-poor plasma (PPP) exhibited no biological activity, with AF cells demonstrating greater responsiveness than NP cells [118]. Subsequent studies elucidated the role of transforming growth factor-β1 (TGF-β1) as a key mediator of PRP activity [119]. In fact, Chen et al. reported that PRP significantly upregulates ECM-related genes, including COL II and ACAN, and promotes proteoglycan accumulation in human NP cells in a dose-dependent manner [119]. These regenerative effects were mediated via phosphorylation of the Smad2/3 signalling pathway [119]. In an ex vivo porcine IDD model, PRP treatment—alone or combined with mesenchymal stem cells (MSCs)—reduced apoptosis, enhanced NP cell viability, increased ECM gene expression, and promoted MSC differentiation toward a chondrogenic phenotype [120]. Beyond anabolic effects, PRP exerts anti-inflammatory actions relevant to IDD pathology. Inflammatory models using IL-1β, TNF-α, or lipopolysaccharide demonstrated that PRP restored ECM gene expression and suppressed catabolic enzymes and inflammatory mediators, including MMPs and COX-2, in both NP and AF cells [121]. These findings suggest that PRP may counteract cytokine-induced degeneration by modulating inflammatory signalling cascades [121]. However, divergent outcomes have been reported depending on PRP composition. Leukocyte-rich PRP (L-PRP) has been associated with increased levels of IL-1β and TNF-α, activation of the NF-κB pathway, enhanced catabolism, and reduced ECM synthesis [122]. In contrast, leukocyte-poor PRP (P-PRP) consistently promoted MSC proliferation and differentiation into NP-like cells without inducing inflammatory or catabolic responses [123]. These observations underscore the importance of leukocyte exclusion in optimizing PRP formulations for disc regeneration.

In summary, in vitro and animal models suggest that PRP enhances IVD cell proliferation, ECM metabolism, and cell survival while exerting anti-inflammatory effects that may be beneficial, especially in early-stage IDD [110]. PRP-mediated disc regeneration is closely linked to TGF-β/Smad signalling and MSC differentiation toward an NP phenotype [123]. Optimization of PRP composition, particularly leukocyte content and concentration, remains critical for maximizing therapeutic efficacy and warrants further investigation.

#### 5.2.2. Clinical Evidence

Across available clinical studies, biologic augmentation with PRP showed beneficial effects in terms of pain relief of IDD syndromes. In a randomized controlled trial (*n* = 30) for single-level lumbar disc herniation, transforaminal epidural PRP produced statistically and clinically meaningful reductions in leg pain at 6, 12, and 24 weeks and improved function by 24 weeks, with no adverse events [124]. Interestingly, clinical outcomes were reported to be superior to triamcinolone [124]. Similarly, in a larger prospective randomized controlled study (*n* = 124) comparing ultrasound-guided transforaminal PRP compared with steroid injection for lumbar disc herniation, the PRP group demonstrated a sustained improvement throughout the first year of follow-up in pain, function, and health-related quality-of-life domains [125]. Moreover, the nerve conduction was demonstrated by the neurophysiologic improvements in F-wave parameters at 1 year [125]. For discogenic low back pain treated via intradiscal administration, a double-blind randomized controlled study (47 analyzed; 92% follow-up) found that a single intradiscal PRP injection yielded greater short-term improvements versus control over 8 weeks in pain, function, and patient satisfaction [126]. A separate intradiscal PRP releasate (PRPr) clinical cohort with 48-week follow-up reported significant reductions in current/best/worst NRS pain and improved SF-36 pain scores (all *p* < 0.001), meeting predefined MCID thresholds [127]. Long-term follow-up of participants from a prior intradiscal PRP trial suggested durability of the procedure, with statistically and clinically significant improvements in pain and function at a mean of 6.6 years (5–9-year range), although ~29% underwent surgery at treated levels during follow-up [128]. Finally, an earlier randomized trial of intradiscal PRPr found significant improvement in low back pain and disability at 12 months despite no meaningful change in radiographic parameters or the prevalence/type of key MRI phenotypes (e.g., Modic changes, disc bulge, HIZ) [129]. Interestingly, a further evaluation of the analyzed cohort allowed the authors to identify multiple target discs and baseline posterior HIZs as predictors of a negative outcome [129]. The available clinical evidence on PRP demonstrates several strengths, including multiple randomized controlled trials and consistent findings of pain reduction and functional improvement. PRP is also characterized by a favourable safety profile and minimally invasive administration. However, significant limitations exist, particularly the lack of standardization in PRP preparation (e.g., leukocyte content, platelet concentration), variability in injection techniques (intradiscal vs. epidural), and heterogeneity in patient selection criteria. Furthermore, most studies focus on short- to mid-term outcomes, with limited evidence regarding long-term structural regeneration. Overall, PRP appears to provide consistent symptomatic relief, particularly in early-stage degeneration, although its regenerative potential remains uncertain.

Strength of evidence: moderate (consistent clinical benefit but limited by heterogeneity and lack of standardization).

## 6. Clinical Perspective

From a clinical perspective, orthobiologic therapies seem to offer a minimally invasive alternative for patients with discogenic low back pain who do not respond to first-line conservative treatment (NSAIDs, analgesics, physical therapy). Patient selection remains a critical issue: disease stage, disc viability, and symptoms still play a central role in treatment decision-making. PRP use has the greatest amount of literature supporting its use in patients with early-stage disc degeneration (Pfirrmann grade II–III) and predominant discogenic pain without structural collapse. On the other hand, MSC- based therapies might be considered in patients with more advanced disease (in terms of both clinics and disc degeneration) but still viable discs. Another key factor is harvesting in early stages. PRP could be a more suitable option due to the easy sampling and processing of patients’ blood; while MSCs harvesting, both from adipose tissue and bone marrow, represents a more laborious procedure that may be worth it in more advanced stages of the disease.

**Figure 3:**
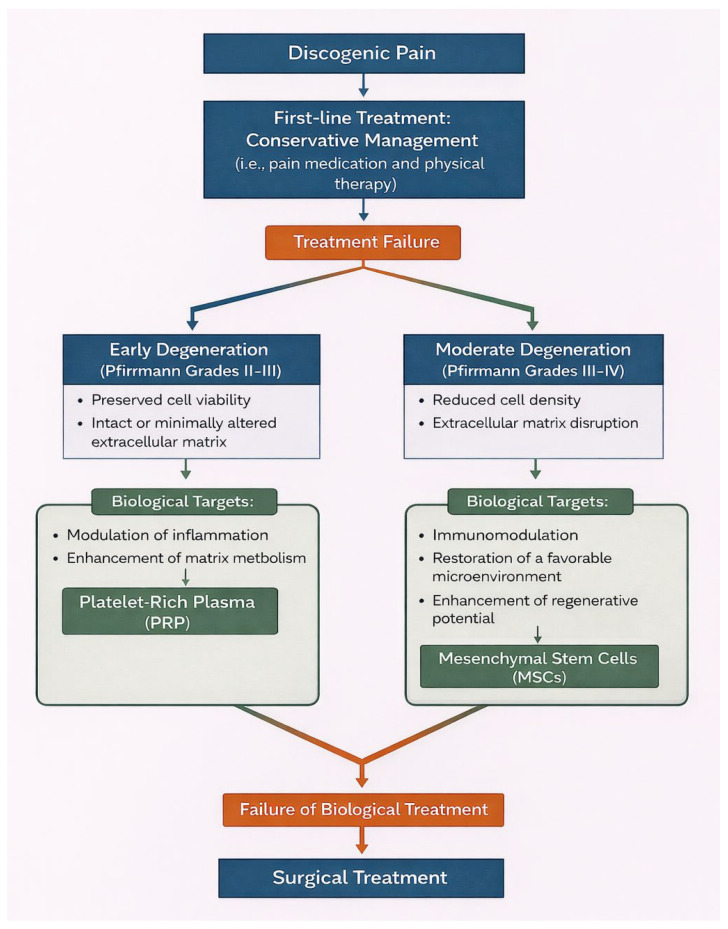
Proposed stepwise clinical approach for intervertebral disc degeneration (graphics realized with AI tool).

Based on the current evidence, a stepwise clinical approach is proposed: (1) first-line conservative management with pain medications and physical therapy; (2) PRP injections in early-stage degeneration with persistent pain; (3) MSCs-based therapies in selected patients with moderate degeneration; (4) surgical intervention when appropriate (Figure 3).

In our opinion, orthobiologics should not be intended as a standalone treatment but rather as a part of a multimodal strategy that includes rehabilitation in order to further improve spine biomechanics.

A summary of proposed indications, mechanisms, and clinical applications is provided in Table 1.

**Table 1:** Comparison between platelet-rich plasma (PRP) and adipose-derived mesenchymal stem cells (AD-MSCs) in intervertebral disc degeneration (IVDD), summarizing mechanisms of action, clinical indications, therapeutic goals, indications, and main advantages and limitations [106,107,108,109,124,125,126,127,128,129].

Therapy	Mechanism of Action	Primary Therapeutic Goal	Proposed Indication	Typical Pfirrmann Grade	Route of Administration	Advantages	Limitations
MSCs	Cell differentiation, paracrine signalling, immunomodulation, ECM synthesis	Structural regeneration and restoration of disc homeostasis	Chronic discogenic low back pain refractory to conservative treatment	II–IV	Intradiscal injection	Potential structural regeneration; long-term clinical improvement; immunomodulatory effects	Limited cell survival; complex preparation; higher cost
PRP (P-PRP preferred)	Growth factor release (TGF-β, PDGF, IGF-1, VEGF), anti-inflammatory and anti-apoptotic effects	Biological stimulation and inflammation modulation	Discogenic low back pain, early disc degeneration, lumbar disc herniation	II–III	Intradiscal or epidural injection	Minimally invasive; autologous; low immunogenic risk; simple preparation	Variable composition; primarily stimulatory rather than regenerative
MSC + PRP combination	Synergistic regenerative and stimulatory effects; improved MSC survival and differentiation	Enhanced regeneration and microenvironment optimization	Moderate disc degeneration requiring regenerative and biologic stimulation	II–III	Intradiscal injection	Synergistic effects; enhanced MSC efficacy; improved regenerative potential	Limited clinical data

Abbreviations: PRP, platelet-rich plasma; AD-MSCs, adipose-derived mesenchymal stem cells; IVDD, intervertebral disc degeneration; ECM, extracellular matrix.

## 7. Limitations

This review has some limitations. As a narrative review, it lacks a systematic methodology and a bias risk assessment. The included studies are heterogeneous in design, limiting comparability. Although promising, the current clinical evidence for MSCs and PRP use is still poor, considering small cohorts, non-standardized protocols, and relatively short follow-up. Further studies with wider cohorts are necessary to assess long-term efficacy and further identify those patients who could benefit more from the procedure.

## 8. Conclusions

MSCs and platelet-rich plasma PRP) seem to be promising biologic therapies for IVDD, thanks to their actions on complementary aspects of the degenerative process. MSCs primarily exert regenerative and immunomodulatory effects through paracrine signalling, promoting EMC synthesis, reducing inflammation, and potentially restoring disc structure. Clinical studies demonstrated significant and durable improvements in pain and functional outcomes following intradiscal MSC administration, particularly in patients with early to moderate degeneration.

PRP, in contrast, acts mainly as a biologic stimulator delivering concentrated autologous growth factors that enhance disc cell viability, stimulate matrix synthesis, and suppress inflammatory and catabolic pathways. Clinical evidence supports its efficacy in reducing pain and disability, especially in early-stage degeneration and discogenic low back pain. Leukocyte-poor PRP appears to provide superior regenerative and anti-inflammatory effects compared with leukocyte-rich formulations.

Combining MSC and PRP may offer synergistic benefits by enhancing MSC survival, differentiation, and regenerative potential, thereby optimizing the disc microenvironment.

Overall, current evidence supports the use of PRP primarily for biologic stimulation in early degeneration and MSCs for regenerative purposes in more advanced but still viable discs, while combined approaches may further improve outcomes in selected cases.

## Data Availability

Data sharing is not applicable.
